# Radiation drives tertiary lymphoid structures to reshape TME for synergized antitumour immunity

**DOI:** 10.1017/erm.2024.27

**Published:** 2024-10-23

**Authors:** Shuling Li, Kuifei Chen, Zhenwei Sun, Meng Chen, Wenhu Pi, Suna Zhou, Haihua Yang

**Affiliations:** 1 Taizhou Hospital, Shaoxing University, Taizhou, Zhejiang, China; 2 Key Laboratory of Radiation Oncology of Taizhou, Radiation Oncology Institute of Enze Medical Health Academy, Department of Radiation Oncology, Taizhou Hospital Affiliated to Wenzhou Medical University, Taizhou, Zhejiang, China

**Keywords:** immune cells, radiation, tertiary lymphoid structure, tumour microenvironment, Immunotherapy

## Abstract

Radiotherapy (RT) plays a key role in the tumour microenvironment (TME), impacting the immune response via cellular and humoral immunity. RT can induce local immunity to modify the TME. It can stimulate dendritic cell maturation and T-cell infiltration. Moreover, B cells, macrophages and other immune cells may also be affected. Tertiary lymphoid structure (TLS) is a unique structure within the TME and a class of aggregates containing T cells, B cells and other immune cells. The maturation of TLS is determined by the presence of mature dendritic cells, the density of TLS is determined by the number of immune cells. TLS maturation and density both affect the antitumour immune response in the TME. This review summarized the recent research on the impact and the role of RT on TLS, including the changes of TLS components and formation conditions and the mechanism of how RT affects TLS and transforms the TME. RT may promote TLS maturation and density to modify the TME regarding enhanced antitumour immunity.

## Introduction

Immunotherapy has sparked an unprecedented revolution in clinical cancer treatment due to its optimistic prognosis (Ref. 1). It has significantly improved the efficacy of treatment in numerous solid tumours and has been progressively incorporated into various cancer treatment guidelines. However, due to primary and secondary immune resistance, only part of patients can benefit from immunotherapy (Refs 2, 3). While the combination therapy, such as immunotherapy combines with other therapies such as radiotherapy (RT), antiangiogenic drugs and neoadjuvant chemotherapy, can significantly decrease the immune resistance, according to accumulated clinical studies (Ref. 4). Recent studies showed that combining immunotherapy with RT can enhance the treatment effect, antitumour response and resistant prevention development in tumour cells (Ref. 5). RT exhibits a modulating effect on the antitumour immune response, both locally and systemically. It has been intensively observed that the potential synergistic effect of RT in combination with immunotherapy improved more cancer control than RT alone in treating many human malignancies (Refs 6, 7, 8). However, the exact mechanism by which RT enhances the infiltration of local T cells, influences the aggregation and differentiation of other immune cells to modify the tumour microenvironment (TME), and indirectly affects systemic antitumour immunity is not fully clear. TME is inconsistent, dynamics and transformation during tumour initiation, progression and treatment, and immunosuppressive TME formation are necessary for immunotherapy resistance.

Tertiary lymphoid structure (TLS) can be formed in certain chronic inflammation, which is a vague concept first proposed in 2009 (Ref. 9). Schumacher *et al*. defined the cellular composition of TLS in cancer, including B cells, T cells, dendritic cells (DCs), follicular dendritic cells (FDCs), follicular reticular cells (FRCs) and high endothelial vein (HEV) (Ref. 10). Mature TLS consists of T cells, B cells, DCs and germinal centres, similar to secondary lymphoid organs (SLOs). TLS is a group of immune cells that locates the tumour's periphery or centre, and is being intensively researched. CD4 + and CD8 + T lymphocytes, B cells, fibroblasts, plasma cells, macrophages and dendritic cells may be present in these structures (Refs 11, 12, 13, 14). Furthermore, TLS contains high endothelial veins (HEVs), which act as a vascular system to maintain connections with related immune cells. Nonetheless, the existence of immune cells in the TME is crucial for local RT; therefore, how does RT regulate tumour development via an antitumour immune function? And how RT influences the TLS remains uncertain. RT affects the role of TME in anti-tumour immunity, which requires further investigation.

Our knowledge of the local antitumour immune response has much space for expansion. TLS is a component of the antitumour immune response; however, its function can be influenced by numerous factors, such as local secretion of inflammatory factors, cytokines, other immune populations, local vascular and epithelial cell signals and therapeutic approaches like chemotherapy and RT. In this review, we first discussed the synergistic effect of RT on antitumour immunity, then we discussed how RT exerts an antitumour effect by reshaping the TME, finally, we discussed TLS as a unique structure within the TME. More, we discussed the potential influence of RT on the formation of TLS to reshape the TME.

## Synergistic effect of RT on antitumour immunity

RT may stimulate the systemic immune response by infiltrating CD8 + T cells and modifying the immunosuppressive microenvironment in unirradiated subcutaneous tumour lesions (Ref. 15). Most patients with T3/T4 pancreatic cancer receive induction chemotherapy for a median of only 4 months (0.5–18.4). The median OS of patients receiving ablative RT was 26.8 months. Patients with inoperable pancreatic cancer are prone to survive after receiving ablative RT (Ref. 16). As matter of fact, RT is the current treatment for glioblastoma. Quetiapine acts as a dopamine receptor antagonist to reduce the self-renewal of glioma cells Quetiapine combined with RT can prolong the survival of glioma mice (Ref. 17). Besides, RT combined with gene-mediated cytotoxic immunotherapy for adult glioblastoma demonstrated safety and potential efficacy, according to a Phase I clinical trial study (Ref. 18). Compared to untreated animals, RT substantially suppressed the growth of tumours in mice with triple-negative breast cancer (Ref. 19). Furthermore, immunotherapy combined with RT can augment the antitumour immune response. Compared to immunotherapy alone, combining RT and immunotherapy significantly reduced tumour growth and prolonged overall survival (OS) in mice models (Refs 20, 21) Current clinical evidence indicates that RT combined with various immune checkpoint inhibitors (ICIs) can improve patients' OS (Refs 8, 22, 23). RT combined with nivolumab can benefit OS and progression-free survival (PFS) (Ref. 24). In a retrospective study of patients with late-stage NSCLC, RT coupled with ICIs can enhance PFS and OS (Ref. 6). One study found that the combination of ICIs and RT can increase the rate of local control, nevertheless, it decreases disease-free survival (Ref. 25). Therefore, RT may improve antitumour immunity.

Alternatively, RT can induce systemic immune changes via local immune regulation. RT inhibits the growth of distant malignancies, a phenomenon known as the abscopal effect. Several studies have shown that patients with melanoma and renal cell cancer who receive RT combined with ICIs experience a significant reduction in distant tumours (Refs 26, 27). One study evaluated 16 patients with metastatic tumours, including melanoma, non-small cell lung cancer and renal cell carcinoma. The median time to disease progression after anti-PD1 treatment alone was 3 months, PFS was significantly longer after combined RT, and remarkably, one person achieved a significant complete response lasting >6 months. Three melanoma patients had an abscopal effect, an incidence of 18.7% (compared with 25% of melanoma patients) (Ref. 28). OS was 10 months when immunotherapy was administered alone and 19 months when immunotherapy was combined with RT (*P* = 0.01). Additionally, the complete response rate for RT and immunotherapy increased from 6.5 to 25.7% (Ref. 29). This distancing effect depends on the presence of T cells, indicating that RT can enhance the immunogenicity of tumours and could be used to improve the efficacy of immunotherapy (Ref. 30).

In conclusion, RT can improve antitumour immunity, and this effect is amplified when combined with immunotherapy. Local immune regulation and subsequent induction of systemic immunity after RT reshaping the TME are primarily responsible for the control effect of RT on local and distant malignancies. RT can enhance tumour immunogenicity and modify TME's efficacy. It may be an essential mechanism for RT-induced immune synergism.

## RT-mediated reshaping of tumour TME

RT may stimulate tumour cells to secrete chemokines, thereby facilitating the conditions for the recruitment of immune cells. Several studies indicated that a combination of RT and anti-PD-L1 antibodies increases CD8 + T cell infiltration in mice models (Refs 31, 32, 33). RT increases T-cell infiltration at local tumour sites, and enhances distant effects when combined with ICIs (Ref. 34). RT increases the release of granulocyte–macrophage colony-stimulating factors (GM-CSF) by stimulating co-stimulatory molecules in T cells (Ref. 35). RT promotes tumour cell recognition by cytotoxic T cells by predominantly elevating the expression of major histocompatibility complex I (MHC class I) (Ref. 36). RT stimulated antigen-specific CTL lysis of tumour cells by modulating the Fas/Fas pathway (Ref. 37). RT produces cytokines (IL-6, IL-1B and TNF-*α*) that encourage T cell's function, expansion and differentiation (Refs 38, 39, 40). Numerous investigations have demonstrated that RT can induce T cell homing and infiltration into the TME (Refs 34, 41, 42, 43). RT can increase natural killer (NK) cells' cytotoxicity of tumours and promote their entry into TME (Refs 44, 45). B cells play an essential role in humoral immunity and antigen presentation. Studies have demonstrated that B cell activation and humoral immunity are the primary mediators of the antitumour effects of RT combined with immunotherapy (Refs 46, 47). Existing clinical data also suggested that the number of B cells in TME elevated considerably following RT (Ref. 48). RT assists the infiltration of CD8 + T cells in esophageal squamous cell carcinoma, which may depend on type I IFN, and increases the expression of CXCL10 and CCL5 by stimulating the intrinsic cGAS-STING pathway in tumour cells (Ref. 49).

Moreover, RT may induce immunosuppressive alterations in the TME, including increased inhibitory cells, such as Tregs and TAMs. RT could activate immunosuppressive signalling pathway by the induction of HIF-1a, which stimulates PD-L1 expression in tumour cells, tumour-associated macrophages and dendritic cells (Ref. 50). The expression of IDO after RT correlates with the increase of TAMs or MDSCs, and inhibiting IDO can improve the effectiveness of RT (Ref. 51). Some studies have demonstrated a negative correlation between macrophages and the survival of solid malignancies (Refs 52, 53, 54). This macrophage phenotype is mainly M2 macrophages. The number and function of T, B, NK and antigen-presenting cells can be increased by RT, it can induce immunogenic cell death or MHC upregulation via immune cells' interferon and toll-like receptor signalling (Refs 55, 56, 57). RT can stimulate the release of tumour antigens, allowing for the presentation of APCs and activation of CD8 + cells (Refs 57, 58). In malignancies, RT can induce an antitumour immune response. Local and systemic responses constitute this antitumour immunity.

The TME undergoes dynamic changes during antitumour therapy, with RT primarily causing TME remodelling via its impact on cellular components, cytokines and specific pathways. Numerous indicators suggest that RT reshaped TME. However, the internal precise mechanism is not yet well-defined, as we can only observe that RT modulates particular components of TME, and it is unidentified how these components play a synergistic role.

## TLS is a unique structure of TME with immune modulating component

### Definition and components of TLS

Numerous cytokines, growth factors, extracellular matrix and different types of cells including endothelial cells, immune cells, etc, are present in tumour immune microenvironments. They surround tumour cells and are nourished by blood vessels. It has a substantial effect on the therapeutic effectiveness of tumours. Immune cell aggregates in non-lymphoid tissues and generate TLS. Presently, they are associated with chronic inflammation, including autoimmunity, chronic infections and cancer. TLS is prevalent in the inner regions of B cells and T cells in the periphery. HEVs are speculated to promote lymphocyte recruitment as the particular vascular system in TLS. Dendritic cells, macrophages and other immune cells may also be incorporated. T cells in TLS are primarily CD3 + T cells, including CD4 + T cells and CD8 + T cells. In 2011, L de *et al*. showed that most T cells were CD62L + , primarily CD4 + memory phenotype in TLS in human lung cancer (Ref. 59). Furthermore, TLS contains CD103 + T cells (Refs 60, 61). Germain *et al*. demonstrated that the density of follicular B cells and mature dendritic cells can assess a patient's optimal clinical prognosis (Ref. 62). TLS composition may differ based on location. It was found that the superficial layer of TLS contained substantially more T helper cells and early TLS than the deep layer (Ref. 14).

TLS has been found in various human tumours including lung, melanoma, breast and colorectal cancers, however, it is uncommon in mouse tumour models. In patients with grade 1 or 2 non-functional pancreatic neuroendocrine tumours, TLS consists primarily of B cell follicles and T cell regions with dendritic cells (Ref. 63). A study revealed that CD3 + T cells, CD20 + B cells, CD8 + T cells, CD208 + dendritic cells and CD21 + follicular dendritic cells located in TLS of hepatocellular carcinoma (Ref. 12). Moreover, some studies implied that antitumour plasma cells may be located in TLS (Ref. 64). TIM4 + macrophages (MΦ) are present in the cancer-associated T-cell region of TLS. TLSTIM4 + MΦ is enriched in tumours with elevated CD8 + T cell infiltration, associated with antitumour immunity (Ref. 65). A TLS-related immune cell infiltration study found that CD3 + T, CD8 + T and CD20 + B cell infiltration elevates in patients with gastric cancer, whereas CD68 + cell infiltration is limited (Ref. 66). TLS rich in CD20 + B cells, CD8 + T cells, CD4 + T cells and CD38 + plasma cells are found in endometrial carcinoma (Ref. 13). TCF1/TCF7 + T cells showed a significant association with TLS in a model of oral cancer (Ref. 67). CD20 + CD22 + ADAM28 + B cells are present in various tumour-associated TLS (Ref. 68). The density of CD8 + T cells and CD20 + B cells was high in TLS-positive tissues (Ref. 69). TGF-*β*-mediated SATB1 inhibition promotes T cell differentiation to Tfh and further promotes TLS formation in ovarian tumours (Ref. 70). TLS mainly comprises B cells, T cells, macrophages, DCs, FDCs (follicular dendritic cells), and HEVs.

**Figure 1.**
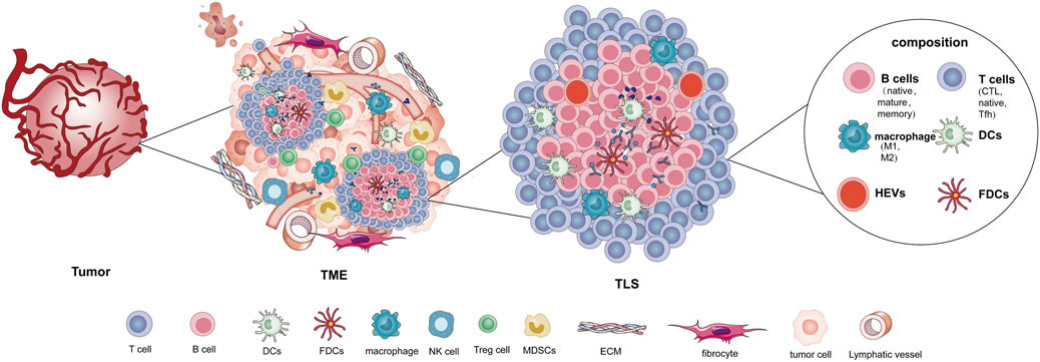
TLS is a special structure in TME. TLS is mainly composed of B cells (native, mature, memory), T cells (CTL, native, Tfh), macrophage (M1, M2), DCs, FDCs and HEVs.

TME refers to the surrounding microenvironment in which tumour cells exist, including surrounding blood vessels, lymphatics, immune cells, fibroblasts, MDSC, various signalling molecules and extracellular matrix (ECM). TME is a complex environment that assists in the survival and development of tumour cells, particularly the immune cells. As a cluster of immune cells, TLS is an essential immune modulating component of the TME (Fig. 1). TLS regulation may play a significant role in TME reconfiguration.

### Role of TLS in tumour progression and prognosis

Reduced TLS formation in ALK + lung adenocarcinoma is closely correlated with tumour progression and may account for reduced immunotherapy response (Ref. 71). The high-density of Treg cells in TLS correlates with decreased survival in NSCLC patients (Ref. 72). Relapsed patients with advanced colorectal cancer have substantially elevated levels of Th (T helper)-type TLS (Ref. 73). One study revealed that breast cancer patients with peritumoral TLS had worse DFS and OS than those with TLS (-) (Ref. 74). TLS was associated with a poor prognosis in kidney clear cell carcinoma, however, it showed an improved prognosis in bladder cancer, according to a comparative analysis (Ref. 75). Early liver cancer can result in immature TLS formation and local immune activation, while promotes immune evasion and tumour progression (Ref. 76). Particular reasons require further investigation. Although there are few studies on TLS and tumour progression, TLS may play numerous functions in various cancer types, particularly in genetic mutations or advanced tumours, which may be one of our future research directions.

Previous research showed that TLS is an immune cell aggregation in the TME. Schumacher summarized that TLS possesses multiple advantages over peripheral circulation lymphocytes or tumour infiltration lymphocytes in the context of antitumour immunity (Ref. 10). **First speed**: priming T and B cells at the TLS may shorten the time required to generate immune responses because it bypasses the immune cells to and from the SLOs. **Second efficiency**: the formation of local germinal centres can enhance the efficiency of immunotherapy by initiating both humoral and cellular immune responses. **Third control**: direct exposure of TLS-associated immune cells to the TME may alter immune response reception of particular output signals. **Fourth survival**: the interaction between TLS-associated effector T cells and APCs promotes patients' survival. Some studies suggested a connection between profuse TILs and TLS (Refs 77, 78). Researchers also noted that mature TLS may activate lymphocytes to perform an antitumour immune role (Refs 79, 80). A higher TILs number in TLS was associated with a better prognosis (Refs 81, 82, 83). The number of TLS in a breast cancer metastatic site is proportional to the number of TILs (Ref. 84). Thus, TILs were substantially correlated with TLS and had a favourable prognosis. When examining the relationship between various therapies and TLS, we can also investigate the relationship between TILs and multiple treatments.

Numerous studies examined the association between TLS and good tumour prognosis in melanoma, lung cancer and colorectal cancer. TLS is essential for initiating and maintaining local and systemic T and B cell antitumour responses, stimulating cellular and humoral immunity channels and increasing antitumour immune response. Currently, TLS has been identified in nearly all solid tumours and has been associated with clinical outcomes in patients with non-small cell lung cancer (Refs 80, 85, 86), pancreatic neuroendocrine tumours (Ref. 63), breast cancer (Ref. 87), gastric cancer (Refs 60, 78), esophageal squamous cell carcinoma (Ref. 88), early oral tongue squamous cell carcinoma (Refs 89, 90, 91), cutaneous angiosarcoma (Ref. 92), epithelioid pleural mesothelioma (Ref. 93), hepatocellular carcinoma (Refs 94, 95, 96), perihilar cholangiocarcinoma (Ref. 97) and endometrial cancer (Ref. 98), displayed in Table 1. Simultaneously, the prognosis of tumour patients changes according to the presence, maturity, location, and high or low TLS signature (Table 1). Most studies have found that TLS is associated with a favourable prognosis; however, TLS may play distinct roles in various regions of hepatocellular carcinoma. In intra-tumoral tissue, the prognosis of DFS in the TLS + group was better. However, in peritumoral tissues, the TLS-group had better OS and DFS outcomes than the TLS + group (Ref. 96). Another study demonstrated that intra-tumour TLS is substantially associated with improved RFS and DFS (Refs 94, 95). Another study revealed that TLS did not affect the OS of patients with liver cancer but significantly affected RFS (Ref. 81).

**Table 1.** Initial studies discerning the effect of TLS on various cancer patients' outcomes

Tumour types	TLS markers	Outcomes	TLS classification	No. of patients	References
Lung cancer	TMB、CXCL13、CCL19、CCL21	Better RFS	TLS + 、TLS-	112	(Ref. 86)
Gastric cancer	CD20 + B、CD3 + T、CD21 + FDCs	Better OS	TLS + 、TLS-	846	(Ref. 78)
Early oral tongue squamous cell carcinoma	NA	Better OS、disease-specific survival	TLS + 、TLS-	310	(Ref. 89)
Cutaneous angiosarcoma	CD20, CD3 and PD-1	Better disease-specific survival	TLS + 、TLS-	31	(Ref. 92)
Epithelioid pleural mesothelioma	CD20、CD3	Long OS	TLS + 、TLS-	129	(Ref. 93)
Hepatocellular carcinoma	Increase: CD3 + , CD8 + , CD20 + Decrease: Foxp3 + and CD68 + cells、PD1 + , TIM3 + and LAG3 +	Decrease early tumour recurrence，OS-independent	TLS + 、TLS-	462	(Ref. 95)
Pancreatic neuroendocrine tumours	CD4 + T cells, CD8 + T cells, CD20 + B cells and CD45RO + memory T cells	Better OS、RFS	TLS + 、TLS-	307	(Ref. 63)
Esophageal squamous cell carcinoma	CD45 + leucocytes, CD20 + B cells, CD4 + and CD8 + T cells, and CD11c + dendritic cells	Better DFS、OS	TLS + 、TLS-	185	(Ref. 88)
Hepatocellular carcinoma	NA	Better OS、DFS、RFS	Intra-tumoral (iTLS) TLS + and iTLS-	364	(Ref. 94)
Lung cancer	CD8 + 、Foxp3 + cells	Better OS、DFS	Mature TLS、immature TLS	218	(Ref. 80)
Endometrial cancer	Bcl6、CD20、CD4、CD8、L1CAM	Lower recurrence，Lower 5-year risk of recurrence	Mature TLS	660	(Ref. 98)
Early oral tongue squamous cell carcinoma	CD3, CD20, CD21, Bcl2 and Bcl6	Better DFS、OS	High-mature TLS、Low-maturity	97	(Ref. 91)
Lung cancer	NA（not applicable）	Better OS、PFI （progression-free interval）	TLS signature high、TLS signature Low	515 in TGCA cohort	(Ref. 85)
Breast cancer	NA	Better DFS、OS	High-TLS signature、low-TLS	3893 in TGCA	(Ref. 87)
Gastric cancer	CD103 + T、CD20 + cell	Better OS	TLS high、TLS low	19	(Ref. 60)
Early oral tongue squamous cell carcinoma	IL7、LTB、CXCL13	Better OS、DFS	Intra-tumoral TLS and peritumoral TLS	65	(Ref. 90)
Hepatocellular carcinoma	CD15，CCL2,CCL4,CCL5,CCL8,CCL18,CCL19,CCL21,CXCL9, CXCL10, CXCL11 and CXCL13	Peritumoral：worse OS、DFS Intra-tumoral：better DFS	Peritumoral 、Intra-tumoral TLS	170	(Ref. 96)
Perihilar cholangiocarcinoma	CD20, CD21, CD8 and PNAD	Better OS、RFS	Intra-tumoral secondary follicle-like TLS	93	(Ref. 97)

TLS can be categorized as mature TLS, immature TLS and negative TLS. Compared to immature TLS, the proportion of proliferating B cells, and CD4 + T cells increased in mature TLS, and B memory cells and Th17 cells increased in mature TLS. In esophageal cancer patients, mature TLS in conjunction with elevated CD8 + T cell infiltration is correlated with optimal survival, and the prevalence of mature TLS is an independent prognostic factor (Ref. 99). In contrast, patients with mature TLS had prolonged survival, activated CD4 + memory cells, primitive B cells and NK cells than those with early-stage TLS (Ref. 100). Similarly, patients with esophageal squamous cell carcinoma who received neoadjuvant chemotherapy had a superior prognosis in the mature TLS high-density group than in the mature TLS low-density group (Ref. 101). In colorectal cancer, *Helicobacter hepaticus*-specific Tfh cells may facilitate TLS maturation, and further enhance antitumour immunity by promoting TLS maturation. This antitumour effect is surprisingly dependent on CD4 + T cells, B cells and natural killer cells, rather than CD8 + T cells (Ref. 102). The analysis of 33 cases of colorectal cancer revealed that the expression of IL-36*γ* was correlated with the infiltration of CD4 + T cells and the enhancement in B cell density in TLS (Ref. 103). It has been found that low-dose RT combined with PD-1 inhibitors increased both the quantity and maturity of TLS in patients with lung adenocarcinoma (Ref. 104).

TLS can be classified as A, B and C grades based on the density of immune cells. The OS of patients with grades C or B improved significantly. TLS and CD20 + B cells were considerably higher in triple-negative breast cancer patients with high-density plasma cells than those with low-density plasma cells (Ref. 105). TLS can also be divided into internal and peripheral tumours based on location. It has been revealed that intra-tumour TLS may have a better OS (Ref. 106). Another study also showed that tumour-associated B cells are predominantly present in TLS (Ref. 107). Both T follicular helper cells and regulatory T cells were significantly elevated in intra-tumoral TLS relative to the peritumoral area, and patients with intra-tumoral intrahepatic cholangiocarcinoma had a better prognosis (Ref. 108).

TLS has the potential to stimulate antitumour immunity. TLS can improve the immune response in many solid tumours, and the level of TLS may be a predictor of immunotherapy efficacy. It has been suggested that B cells in TLS can act as APC to prompt cytotoxic T cells (Ref. 109). In rhabdomyosarcoma, T cells in TLS may increase antitumour response (Ref. 110). Intra-tumoral TLS was linked to a decreased risk of early recurrence in HCC patients undergoing surgery. It is suggested that TLS in tumours may indicate sustained and effective antitumour immunity (Ref. 111). Studies also demonstrated that in hepatocellular carcinoma, peritumoral TLS showed significantly higher immune infiltration and positive immune response. Patients with a high peritumoral TLS density have excellent clinical outcomes (Ref. 112). Intra-tumour TLS is associated with the response to antitumour immunity in renal cell carcinoma (Refs 64, 113).

In summary, the presence of TLS in TME has a significant association with the clinical prognosis in solid tumours. We can see better OS, RFS and DFS in numerous solid tumours when TLS appears. These outcomes are based on the function of TLS in the TME. Similarly, the location, density and maturation of TLS also influence the outcomes. The relevant results need to be further studied.

## RT Modulates TLS to reshape TME in regulating antitumour immunity

**Figure 2.**
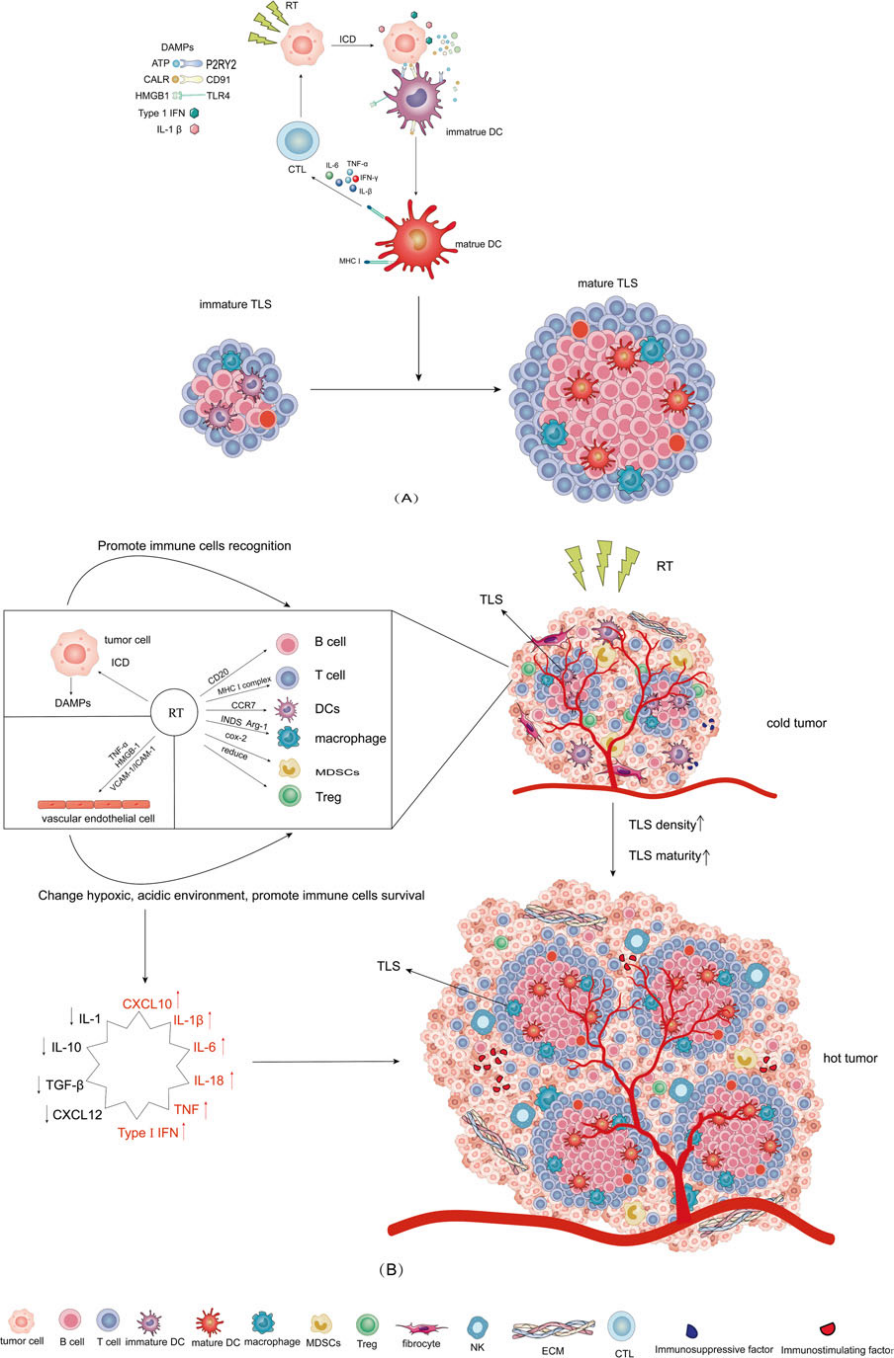
Mechanism of radiation (RT) modulates TLS to reshape TME in regulating antitumour immunity. (A) RT acts on tumour cells via ICD, tumour cell releases DAMPs, which can promote immature DC to mature DC and present associated antigens to cytotoxic T cell. After RT stimulation, immature TLS transforms into mature TLS. (B) RT changes hypoxic, acidic environment and promotes immune cells recognition and survival. It can modulate TLS density and maturity. RT can increase immunostimulating factors such as CXCL10, IL-1J3, IL-6, IL-18, TNF, Type 1 IFN and decrease immunosuppressive factors (CXCL 12, TGF-J3, IL-10, IL-1). Finally realizing the remodelling of TME from cold to hot.

RT primarily modulates cellular components such as a tumour, vascular endothelial and immune cells to reshape TME. Furthermore, RT activates the NF-*κ*B signalling pathway and simulates the release of immune-stimulating factors, such as CXCL10, IL-1*β*, IL-6, IL-18, TNF and type I interferon (Ref. 114). Type I interferons can promote the polarization of tumour-associated macrophages from M2 to M1 (Ref. 115). Moreover, other cytokines can stimulate T cell recruitment, thereby playing an immunostimulatory role. Meanwhile, M2-TAMs release cytokines (including IL-1, IL-6, IL-10 and TGF-*β*), and cancer-associated fibroblasts secrete CXCL12 to attract immunosuppressive cells (Treg and MDSCs), which enables immunosuppressive cells recruitment and effector cells exclusion (Ref. 115). TLS is the primary immune cell colony of the TME. Consequently, based on the above review results, we hypothesized that RT might modulate immune cells in TLS and modify the quantity and function of TLS, to remodel TME from cold to hot (Fig. 2).

### RT regulates non-immune cells in TME

When tumour cells die following exposure to RT, they can change from non-immunogenic to immunogenic, prompting an antitumour immune response referred to as immunogenic cell death (ICD). ICD can produce new antigenic epitopes from dead tumour cells, generate damage-associated molecular patterns (DAMPs), and recruit antigen-presenting cells (APC). APC identifies antigens associated with tumour cells, presents them to T cells, activates adaptive immune responses, and produces durable antitumour immunity. RT induces the formation of reactive oxygen species (ROS), which facilitate the release or exposure of DAMPs (Ref. 116). RT can induce ICDs and change surface markers of tumour cells, including ATP, CALR, HMGB1, Type 1 IFN and IL-1*β* (Ref. 117). Extracellular ATP links to the purinergic receptor P2Y2 (P2RY2) to serve as a ‘find-me’ signal for dendritic cell precursors and macrophages, thus encouraging myeloid cell recruitment to the active ICD site (Ref. 118). In addition, amplifying the ‘eat me signal’ during ICD is crucial for cancer immunotherapy. Some treatments such as radiation therapy and photodynamic therapy can initiate or promote ICD and activate an antitumour immune response (Ref. 119). CALR stimulates LDL receptor-associated protein 1 (LRP1, also known as CD91) on phagocytes to activate antigen-producing cells such as DC and macrophages that promote the clearance of tumour cells (Ref. 120). Extracellular HMGB1 can bind to numerous PRRs expressed by myeloid cells, with the most common mode being Toll-like receptor 4 (TLR4), which stimulates the release of pro-inflammatory cytokines (Refs 121, 122). The immunogenicity of radiation induced ICDs relies on type I IFN signalling (Ref. 123). Anthracyclines induce the endothelial cell pattern recognition receptor TLR3 to activate tumour cells to rapidly generate type I interferon and stimulate the release of CXCL10 (Ref. 124). When ICD arises, the above DAMPs are released, APC (macrophages, DC) are activated and matured, and the secretion of IL-6, IL-1*β*, TNF-*α* and IFN-*γ* by mature DC promotes the differentiation of T cells into CD8 + phenotypes (Ref. 125). Cross-presentation of antigens by DC activates and transforms CD8 + T cells into cytotoxic T lymphocytes. TLS is considered ‘mature’ when at least one CD23 + dendritic cell is present (Ref. 126). RT can promote the development of immature DC into mature DC, so we speculate that RT can also promote the development of immature TLS into mature TLS (Fig. 2A).

Different radiation dose caused varying degrees of damage to tumour vessels. At lower doses (6 or 12 Gy), the vascular bed recovered approximately six days after exposure. When a higher dose of 18 Gy was administered, the tumour's response time was substantially prolonged, and recovery did not commence until the 10th day (Ref. 127). Another study found that irradiation higher than 10 Gy /F may cause significant vascular damage, resulting in decreased blood perfusion, and damage to the microenvironment within the tumour (Ref. 128). High-dose radiotherapy (HDRT) promotes Notch1 signalling pathways and Notch1 expression, and Notch1 activation might defend tumour vessels from HDRT-induced damage (Ref. 129). RT can promote the death of vascular endothelial cells, producing chemokines such as TNF-*α* and HMGB-1 (Ref. 130). RT upregulates adhesion molecules (VCAM-1/ICAM-1 and p/e selection), normalizing the aberrant vascular system and facilitating the recruitment of circulating immune cells (Ref. 115). RT might help initiate and maintain vascular normalization in TME (Refs 41, 131). However, lactic acid accumulation in hypoxia/HIF-1*α*-driven TME can inhibit T cell proliferation, tumour infiltration and cytokine production, suppress the cytotoxic activity of NK and CD8 + T cells, and enhance the number of MDSCs (Refs 132, 133, 134).

### RT and various immune cells in the TLS

RT eliminates tumour cells and induces the release of pro-inflammatory molecules. Necrotic tumour cells generate antigens associated with the tumour. APCs acquire tumour-associated antigens, migrate to draining lymph nodes, and present these antigens to specific T cells via MHC Class I molecules, enhancing T cell activation and proliferation. CD8 + T cells that have been activated can prevent the production of immunosuppressive cells, produce perforin and granzyme, and exert an antitumour effect. What impact does RT have on various immune cell types?

#### T Cells

Numerous studies have demonstrated that RT can promote the infiltration of local T cells for enhanced antitumour effects. RT increased CD45RO + memory T cells and CD4 + regulatory T cells (Ref. 135). Following RT for rectal cancer, the proportion of CD4 + T cells and memory T cells was higher in the response group than in the non-response group, while the proportion of CD8 + T cells and M2 was lower (Ref. 136). RT amplified the percentage of antigen-experienced T cells and effector memory T cells. RT upregulates the tumour-associated antigen-MHC complex, improves antigen-cross-presentation, and numerous studies demonstrate that RT increases T-cell infiltration in tumours (Refs 34, 46, 137, 138).

RT may stimulate T-cell infiltration in many solid tumours such as glioma, pancreatic adenocarcinoma, triple-negative breast and cervical cancers (Refs 139, 140, 141, 142). T cells and macrophages enhanced after RT for melanoma (Refs 143, 144). RT can also increase the intra-tumoral invasion of eosinophilic granulocytes, thereby improving intra-tumoral T cell invasion (Ref. 145). In mouse lung tumour models, RT can initiate and activate antitumour immunity by improving the infiltration of CD8 + and CD4 + T cells (Ref. 146). In RCC patients treated with SBRT, the expression of calreticulin and TAA and the proportion of proliferating T cells increased (Ref. 147). WBRT promotes the expression of the MHC Class I complex and enhances T-cell infiltration in glioma cells (Ref. 148). Ultimately, RT may facilitate the infiltration of T cells into the TME, specifically CD8 + and CD4 + T cells, which are positive prognostic immune cells.

#### B Cells

Lymphocytes contain numerous B cells in antitumour immunity in addition to T cells. Numerous researchers are investigating B cells' function in antitumour immunity, and they speculate that B cells may play a more significant role than is currently believed. Thus, what is the relationship between RT and B cells? Different RT regimens have distinct immune impacts (Ref. 149). Studies have demonstrated that stereotactic body radiotherapy (SBRT) induces tumour infiltration of CD8 + T cells, B cells and macrophages significantly better than large-field RT therapy. RT influences the development of B cells in bone marrow, increasing early and late pro-B cells (Ref. 150). Lower levels of naive and double-negative B cells were observed. However, after hypo-fractionated stereotactic RT, the proportions of MZ-like B cells, transitional B cells, and plasma cells were increased (Ref. 151). In irradiated mice, bone marrow progenitor cells were reduced, however, RT did not affect the development of B-1a cells (Ref. 152). In patients with cervical cancer, the function of B cells was unaffected by RT, which is more radiation-tolerant than other lymphocyte subsets (Ref. 153). In early-stage NSCLC, SBRT may cause immunosuppression and decrease CD3 + , CD4 + , CD8 + , CD19 + , and CD56 + cell counts (Ref. 154). All lymphocytes decreased following RT, and in univariate analysis, lower total B cell counts were linked to ≥ grade 2 RT pneumonia (Ref. 155). Ki67-/DNMT3a + naive B cells, as the largest subgroup of B cells after RT, enhance T-bet expression, related to phosphorylation of p90RSK expression. In vitro, p90RSK activation was also found to upregulate naive B cells (Ref. 156). B cells recovered 180 days after RT, while CD4 + and CD8 + immature T cells remained substantially lower than baseline (Ref. 157). The number of CD19 + B cells decreased after RT and recovered gradually after two months (Ref. 158). B cells are radio-resistant to radiation-induced apoptosis (Ref. 159). Nonetheless, RT encourages the development of memory B cells and antigen-specific B cells (Ref. 160). RT can induce CD20 expression, a common surface antigen on B cells (Ref. 161). In summary, the majority of B cells diminish following RT. Some research suggests that RT stimulates the development of memory B cells and antigen-specific B cells. It can stimulate humoral immunity with antitumour properties.

#### Dendritic Cells

Cross-presentation is a crucial function of dendritic cells in antitumour immunity. Consider the connection between RT and dendritic cells (DC). In RT-tolerant tumours, consumption of Treg can stimulate CD103 + DC activation and increase CD8 + T cells (Ref. 162). Tumour irradiation improves the capacity of DC to acquire tumour antigens, migrate to lymph nodes and deliver treated antigens to T cells (Refs 163, 164). The accumulation of ROS in the RhoA/ROCK1 signalling pathway regulates this homing ability. RT upregulates CD86, CD40, CD80, CXCR4 and CCR7 expression in DC (Ref. 165). It was speculated that RT mediated by ATM/NF-KB increased IL-12 and CCR7 expression and DC migration (Ref. 166). By inducing apoptotic bodies, via the STAT5/Zbttb46 pathway, RT enhances the immune activation potential of DC (Ref. 167). RT can activate derived DC in a mouse lung cancer model (Ref. 168). Briefly, RT may facilitate the activation and homing of DC to lymphoid tissue and initiate an antitumour response.

#### Other immune cells

TME contains numerous immune cells. RT can induce macrophage recruitment in the TME (Refs 169, 170, 171, 172). In liver cancer, RT can recruit macrophages into the tumour (Ref. 173). Nevertheless, it has been suggested that reducing the entrance of macrophages into the TME, and resulting macrophage rejection is a promising strategy for enhancing the tumour's response to RT (Ref. 174). After high-dose irradiation, tumour-associated macrophages were predominantly M2 polarized, and Arg-1, and COX-2 levels were elevated (Refs 175, 176). Another study demonstrates that low-dose RT helps macrophage differentiation towards the iNOS^+^M1 phenotype (Ref. 42). Tumour-associated macrophages are a crucial component of TME, which can be polarized into M1 and M2 types. M1 promotes antitumour activity, while M2 encourages tumour growth. The kind of macrophage infiltration following RT must be investigated further, concerning the cancer treatment prognosis. Additionally, RT decreases the infiltration of immunosuppressive cells including Treg and MDSCs (Ref. 31). Conclusively, RT can transform immunosuppression into immunostimulation.

Numerous studies have demonstrated, as is common knowledge, that RT can enhance the efficacy of immunotherapy. RT combined with immunotherapy can produce a double effect, as the two sensitize and achieve each other. In the era of immunotherapy, the aforementioned studies indicated that TLS in TME enhance antitumour immunity. RT is an essential component of cancer treatment and immunotherapy sensitization. TME is among the significant determinants of antitumour immunity. Consequently, what is the mechanism through which RT influences antitumour immunity? TLS is a unique structure in TME, its numerous components are affected by RT, and RT can promote the local infiltration of immune cells to produce TLS, as described in the preceding section.

First, RT can stimulate the infiltration of T cells into the TME, particularly CD8 + T and CD4 + T cells. Further research is required to determine whether these T cells' aggregation can promote T cell regions' formation in TLS. Second, the alterations in B cells during RT are distinct from those infiltrating T cells. The majority of B cells are reduced after RT. However, some studies show that RT promotes the development of memory B cells and antigen-specific B cells. It might induce humoral immunity to play an antitumour role. Further investigation is required to determine whether RT can boost humoral immunity. Researchers have gradually shifted their attention from antitumour immunity to humoral immunity, and B cells have accomplished specific antitumour immunity effects (Refs 177, 178). Therefore, it remains debatable whether RT can promote the formation potential of B-cell regions in TLS and further discussion is needed. In the end, RT can affect other immune cells other than lymphocytes. RT, for instance, can promote the activation and homing of DC to lymphoid tissue, thereby activating antitumour immunity. We can therefore hypothesize that RT promotes the activation and presentation of DCs in TLS. Additionally, RT can enhance macrophage infiltration, although whether M1 or M2 is unclear.

Irradiated tumour cells can result in ICD. The dead tumour cells secrete DAMPs. These signals recruit antigen-presenting cells, including NK cells, to cross-present antigens to CD8 + T cells, and CTL plays an antitumour immune role. RT facilitates the infiltration of various immune cells into the TME, and normalizes tumour blood vessels. These immune cells assemble to create a particular structure called TLS, which acts more quickly and efficiently against tumours. It is plausible that RT may mediate TLS to reshape the TME, and enhance the antitumour immune environment. The immunogenic effects of RT can transform a ‘cold’ into a ‘hot’ environment, thus sensitizing non-responsive tumours to immunotherapy (Fig. 2B).

## Conclusion and prospect

RT can reshape TME and mobilize the immune response to perform a local and systematical role in the tumour. Overall, we believe that the modulation of RT on TLS is a crucial mechanism for reshaping the synergistic immune effect of TME. RT plays an essential role in activating the immune system via humoral immunity, cellular immunity and other ways, which may be consistent with the local immune activation response formed by TLS, thereby influencing the efficacy of cancer treatment. This review demonstrates that RT mainly affects the formation of TLS, and the formation of TLS can influence systemic immunity via humoral and cellular immunity. Based on preclinical data, TLS may open up new opportunities for more effective immunotherapies; however, we must determine which factors promote TLS.

**Figure 3.**
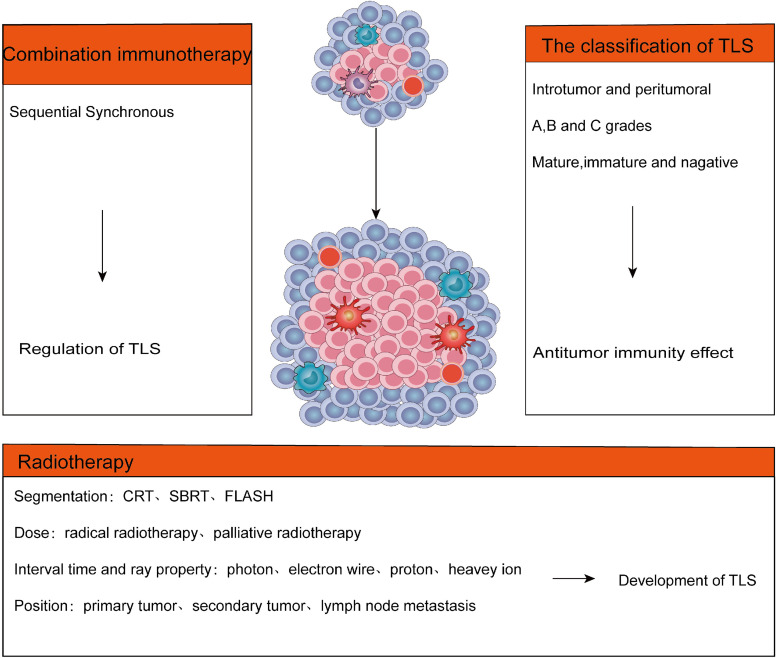
The future perspectives in TLS formation. Effects of different RT dose, segmentation, time and location on TME and TLS. Effect of RT combined with immunotherapy on TLS. Effect of maturity, density and location of TLS on antitumour immunity.

As an aggregate of immune cells that infiltrate tumours, TLS may be an immune-related therapeutic target that leads to the next ‘breakthrough’. The specific pathway of RT regulation on TLS and the endpoint of TLS regulation by RT with distinct doses, segmentation modes, positions and times still require clarification. The various segmentation techniques demonstrate that RT can be subdivided into SBRT, CRT and FLASH. Following the treatment's purpose, RT can be classified as radical or palliative, primarily based on dose. According to RT interval time and ray properties, it can be classified into photons, electron lines, protons and heavy ions. Furthermore, the RT dose is distinct around the tumour and in the centre, and the patient's prognosis of intra-tumour TLS and peritumoral TLS is also variable. TLS has varying species specificity in various tumours, and some solid TME are predisposed to produce this unique structure. The probable effect of RT on TLS can be used as a new research strategy for cancer treatment. In future studies, we need to explore further the following issues: (1) Effects of varying RT time, dose, position and segmentation on the development of TLS; (2) the impact of TLS location, density and maturity on the prognosis of antitumour immunity; (3) RT combined immunotherapy, such as sequential and synchronous, impacts on the regulation of TLS (Fig. 3).
